# Urothelial Bladder Carcinomas with High Tumor Mutation Burden Have a Better Prognosis and Targetable Molecular Defects beyond Immunotherapies

**DOI:** 10.3390/curroncol29030117

**Published:** 2022-02-24

**Authors:** Ioannis A. Voutsadakis

**Affiliations:** 1 Algoma District Cancer Program, Sault Area Hospital, Sault Ste. Marie, ON P6B 0A8, Canada; ivoutsadakis@yahoo.com or ivoutsadakis@nosm.ca; 2 Section of Internal Medicine, Division of Clinical Sciences, Northern Ontario School of Medicine, Sudbury, ON P3E 2C6, Canada

**Keywords:** transitional cell carcinoma, genomics, tumor mutation burden, immunotherapy

## Abstract

**Background:** Urothelial bladder carcinomas had traditionally been difficult to treat cancers, with high morbidity and mortality rates when invasive and metastatic. In recent years, immunotherapy with immune checkpoint inhibitors has improved outcomes in several cancers, including bladder carcinomas. Despite positive overall results, many bladder cancer patients do not respond to immunotherapies. Validated predictive biomarkers of response would advance the selection of patients for these treatments. Tumor mutation burden (TMB) has been suggested as an immunotherapy biomarker and thus delineation of attributes of tumors with a high TMB is clinically relevant. **Methods:** Publicly available genomic and clinical data from the urothelial bladder carcinoma cohort of The Cancer Genome Atlas (TCGA) project are used to analyze characteristics and molecular alterations of the subset of cancers with an increased tumor mutation number compared with those with lower number of mutations. The cut-off for the high mutation burden in the analysis was set at 10 mutations per Megabase (MB). **Results:** In addition to their sensitivity to immune checkpoint inhibitors, urothelial carcinomas with high TMB possess several molecular defects that could be exploited for combinatorial treatments. Compared with bladder carcinomas with low TMB, carcinomas with high TMB display higher prevalence of mutations in tumor suppressor *TP53*, *PIK3CA*, in *FAT4* cadherin and in genes encoding for several epigenetic modifier enzymes. The frequency of mutations in mismatch repair and DNA damage response genes is higher in cancers with high TMB. The group of urothelial carcinomas with high TMB has a better prognosis than the group with low TMB. This improved Overall Survival (OS) stems from improved survival of stage III cancers with high TMB compared with stage III cancers with low TMB, while stage II and stage IV cancers have similar OS, independently of their TMB. **Conclusion:** Differences of the landscape of high and low TMB urothelial cancers provides leads for further pathogenesis investigations and may prove useful for development of combination therapies including immunotherapies with targeted inhibitors.

## 1. Introduction

Urothelial bladder carcinoma is the most prevalent urinary system cancer with an estimated 83,730 new cases diagnosed in 2021 in United States alone and an expected 17,200 deaths [1]. The disease is most common in men for whom it represents the fourth most prevalent cancer after prostate cancer, lung cancer and colorectal cancer. In women, bladder carcinoma represents the twelfth most prevalent cancer [1]. The systemic treatment paradigm of bladder transitional cell carcinoma is currently expanding to include immunotherapy treatments in both the adjuvant and metastatic setting. For the adjuvant therapy of localized disease after surgical resection, treatment with the immune checkpoint inhibitor nivolumab has shown an improvement in disease-free survival [2]. In this study only about two fifths of the patients had received neoadjuvant platinum-based chemotherapy and seemed to derive greater benefit from adjuvant nivolumab (Hazard ratio (HR): 0.52, 95% confidence interval: 0.38–0.71 versus HR: 0.92, 95% confidence interval: 0.69–1.21 in patients who did not receive neoadjuvant chemotherapy). In addition, the benefit of nivolumab was greater in patients with expression of the ligand of the targeted receptor, PD-L1 in more than 1% of tumor cells (HR: 0.56, 95% confidence interval: 0.40–0.80 versus HR: 0.82, 95% confidence interval: 0.63–1.06 in patients with PD-L1 less than 1%) [2]. However, another adjuvant trial with the immune checkpoint inhibitor atezolizumab showed no benefit of this immune checkpoint inhibitor versus placebo [3].

Metastatic invasive bladder carcinomas have a dismal prognosis and until recently palliative platinum-based chemotherapy was the only therapeutic option and remains the first line treatment for eligible patients [4]. Advances in understanding of the molecular pathophysiology of bladder carcinoma led to the introduction of targeted therapies and immunotherapies in later line treatment of the disease [5,6,7]. Immunotherapy with pembrolizumab in patients progressing after platinum-based chemotherapy is more effective than second line chemotherapy with taxanes or vinflunine in prolonging overall survival (OS) and showed a longer duration of response [5]. The benefit of pembrolizumab over chemotherapy was observed in patients across levels of PD-L1 expression. The PD-1 inhibitor nivolumab has been investigated in a phase 2 trial in patients with metastatic urothelial carcinoma progressing after platinum-based chemotherapy [8]. Objective response was 24.4% with responses observed independently of expression of PD-L1 in more than 1% of tumor cells or less than 1% of tumor cells.

Thus, despite the ongoing introduction of immunotherapies in the treatment armamentarium of urothelial carcinomas, there is a paucity of biomarkers to guide optimal therapy in these patients. TMB has been investigated in other cancers as a biomarker of immunotherapies’ response [9]. A tumor agnostic indication for pembrolizumab in cancers with high TMB has been granted by the American Food and Drug Administration (FDA) [10]. The current investigation exploits published genomic data to describe the landscape of urothelial carcinomas with increased tumor mutation burden (TMB) as candidates for immunotherapy. A better understanding of concomitant molecular defects in these cancers could also guide development of combination therapies.

## 2. Methods

The current analysis is based on the published genomic study of bladder carcinomas from The Cancer Genome Atlas (TCGA) [11]. Genomic data from the TCGA bladder carcinoma cohort were interrogated through the cBioportal for cancer genomics platform (http://www.cbioportal.org, accessed 2 December 2021). cBioportal is a user friendly, publicly available platform containing molecular studies and corresponding clinical data from the TCGA network and other groups [12,13]. The cBioportal site, developed by researchers in Memorial Sloan Kettering Cancer Center and currently maintained by a multinational team in United States, Canada, the Netherlands and Turkey, allows for multi-dimensional interrogations of genomic data including point mutations, fusions and copy number alterations. cBioportal links information on molecular lesions in genes included in the original studies with patient clinical and tumor characteristics and survival outcomes [12].

In its studies of various cancers, TCGA employed high throughput platforms for whole exome massively parallel DNA sequencing. Copy number alterations (CNAs) analysis in TCGA cohorts was performed with the GISTIC (Genomic Identification of Significant Targets in Cancer) algorithm [14]. In GISTIC, putative amplification of a given gene is defined as a score of 2 or above. TCGA studies provide an Aneuploidy Score (AS) as a measure of chromosomal instability of each sample. AS is calculated as the sum of the number of chromosome arms in each sample that have copy number alterations (gains or losses). A chromosome arm is considered copy number altered, either gained or lost, if there is a somatic copy number alteration in more than 80% of the length of the arm as calculated by the ABSOLUTE algorithm, which is based on Affymetrix 6.0 SNP arrays [15]. Chromosomal arms with somatic copy number alterations in 20% to 80% of the arm length are considered not evaluable, and chromosomal arms with somatic copy number alterations in less than 20% of the arm length are considered not altered. The cut-off of high mutation number in the current study was set to 10 mutations per Megabase (MB).

The OncoKB knowledgebase (Oncokb.org) is a precision oncology database of cancer related genes, several of which have been manually curated and classified as oncogenes or tumor suppressors [16]. OncoKB also classifies alterations in cancer associated genes according to their putative functional significance and is used in the current study for functional classification of discussed mutations.

For the performance of mutation signature analysis, data on single nucleotide substitutions of TCGA bladder cohort cases were downloaded from the Broad institute firehose.

The Fisher’s exact test or the x^2^ test and the t test, respectively, are used to compare categorical and continuous data. Kaplan-Meier survival curves were constructed and compared using the Log Rank test. All statistical comparisons were considered significant if *p* < 0.05. Corrections for multiple comparisons were made when appropriate with the Benjamini-Hochberg false discovery rate procedure.

No external funding was received from any source for the performance of this study.

## 3. Results

The updated bladder cancer cohort from TCGA consists of 412 patients with mostly stage II to IV disease. The high TMB [>10 mutations/megabase (MB)] group consists of 107 patients (26%), while 304 patients (74%) had a low TMB (≤10 mutations/MB). The mean tumor mutation number/MB in the high TMB group was 19.4 (SD: 14.4) and the mean tumor mutation number/MB in the low TMB group was 4.6 (SD: 2.5). The two groups had no significant differences in the mean age of cancer diagnosis or the percentage of patients older than 65 years-old and in sex (Table 1). The stage of cancer was also similar in the two groups. Most patients in the cohort (97.6%) had not received neoadjuvant therapy.

**Figure 1:**
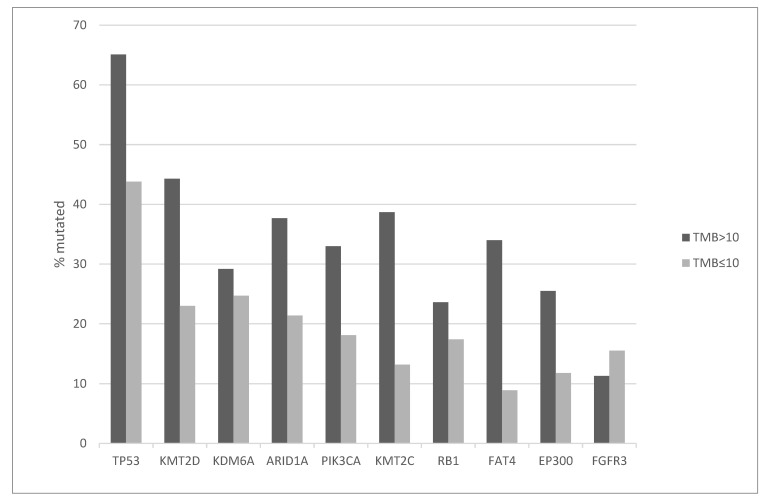
Prevalence of mutations in frequently mutated genes in bladder carcinomas according to TMB groups. High TMB group: TMB > 10 mutations/MB, low TMB group: TMB ≤ 10 mutation/MB. TMB: Tumor Mutation Burden.

The most frequently mutated cancer associated gene in bladder carcinoma is the tumor suppressor *TP53* which displays mutations in almost half (49.3%) of cases in the TCGA cohort. A significantly higher percentage of bladder cancers with high TMB (65.1%) harbor *TP53* mutations compared with the prevalence of 43.8% in the group with low TMB (Fisher’s exact test *p* < 0.0001, Figure 1). Besides *TP53*, the top ten list of mutated cancer associated genes in bladder cancer includes epigenetic regulators *KMT2D*, *KDM6A*, *ARID1A*, *KMT2C* and *EP300*, the gene encoding for the catalytic sub-unit alpha of kinase PI3K, *PIK3CA*, the tumor suppressor RB, the genes encoding for FAT4 cadherin, and for the FGFR3 receptor tyrosine kinase. Among these, mutations in all epigenetic regulators (except mutations in demethylase KDM6A), mutations in *PIK3CA* and in *FAT4* cadherin are significantly more frequent in the high TMB group (Figure 1). In contrast, genes for KDM6A, RB and FGFR3 show similar mutation prevalence in the high and low TMB groups, with no statistically significant differences (Figure 1).

Regarding functional significance of prevalent mutations as evaluated in the OncoKB knowledgebase, 235 of the 237 mutations TP53 present in the whole cohort are listed as likely to be oncogenic. The other five highly prevalent mutated genes in bladder cancers, *KMT2D*, *ARID1A*, *KMT2C*, *PIK3CA* and *EP300* (*FAT4*, although listed as a cancer gene, is not yet curated for functional significance in OncoKB knowledgebase), with difference in prevalence between the high TMB and the low TMB groups, are listed as likely to be oncogenic in 44.4% to 77.8% of cases (Table 2). There are no statistically significant differences in the percentage of likely oncogenic mutations in any of the five genes between the high TMB and the low TMB group, suggesting that the increased occurrence of these mutations in the high TMB group is not solely a result of random increased mutation rates in this group.

**Figure 2:**
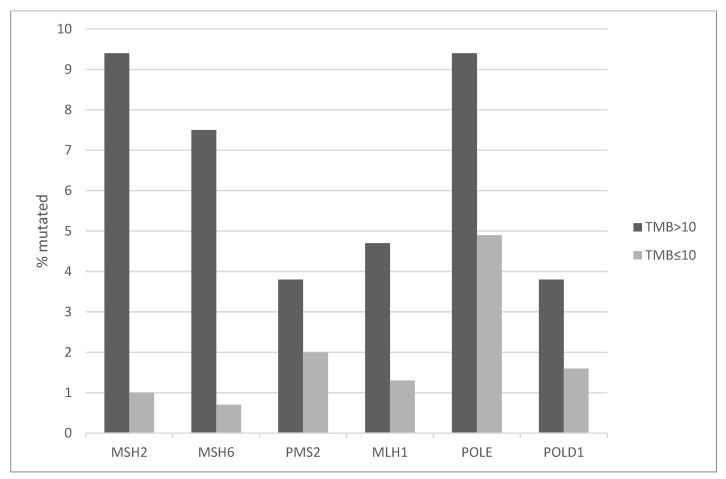
Mutation prevalence in Mismatch Repair related genes and genes encoding for polymerases *POLE* and *POLD1* in bladder carcinomas according to TMB groups. High TMB group: TMB > 10 mutations/MB, low TMB group: TMB ≤ 10 mutation/MB. TMB: Tumor Mutation Burden.

Mutations in Mismatch Repair (MMR) associated genes (*MSH2*, *MSH6*, *MLH1* and *PMS2*) producing Microsatellite Instability or mutations in proofreading polymerases epsilon and delta (*POLE* and *POLD1*) show a low prevalence in bladder carcinomas ranging from 2.2% for *MLH1* and *POLD1* to 6.1% for *POLE*. However, their prevalence is higher in the high TMB group, with *MSH2* and *POLE* mutations observed in 9.4% of cases each (Figure 2). In the high TMB group 29.9% of cases show mutations in one or more of MMR associated or proofreading polymerases genes compared with 10.5% in the low TMB group (Fisher’s exact test *p* < 0.0001). Thus, despite a significant difference between the groups, MMR and proofreading polymerases mutations do occur in a significant minority of samples with low TMB. On many occasions, the functional significance of mutations in these genes is unknown. For example, among the 10 POLE mutations in the high TMB group, one mutation at the hotspot protein position P286 in the exonuclease domain (protein substitution P286R), is listed at the OncoKB knowledgebase as likely to be oncogenic, while the rest are listed as being of unknown significance (Table 3). Similarly, all 15 POLE mutations in the low TMB group are listed in OncoKB as of unknown significance. The sample with the POLE P286R mutation is, as expected by the presence of a deleterious mutation in the proofreading polymerase, ultra-mutated with TMB of 118.6/MB. Another ultra-mutated case with 84.6 mutations/MB contains a POLE mutation of unknown significance (Q1332H) located in an unstructured stretch of the protein between the polymerase domain and a Domain of Unknown Function (DUF). In another example, two of the eight mutations in MSH6 at the high TMB group and none of the two MSH6 mutations in the low TMB group are listed as likely to be oncogenic in the OncoKB database (Table 3).

A detailed analysis of mutations in the 10 ultra-mutated cases of bladder cancers with TMB above 30 mutations/MB discloses that, besides the above-mentioned sample with the likely oncogenic POLE P286R mutation, which also possesses a MSH2 likely oncogenic mutation, no other cases contain likely pathogenic mutations in MMR related genes or the proofreading polymerases (Table 4). Recurring likely pathogenic mutations in ultra-mutated bladder cancers occur in several epigenetic modifiers, such as ARID1A in six cases and in one or more lysine methyltransferases in seven cases. Among genes that are mutated less commonly in bladder cancer, noticeable are likely pathogenic mutations in the nucleotide excision repair helicase ERCC2, observed in three of the 10 cases with TMB above 30 mutations/MB (Table 4).

**Figure 3:**
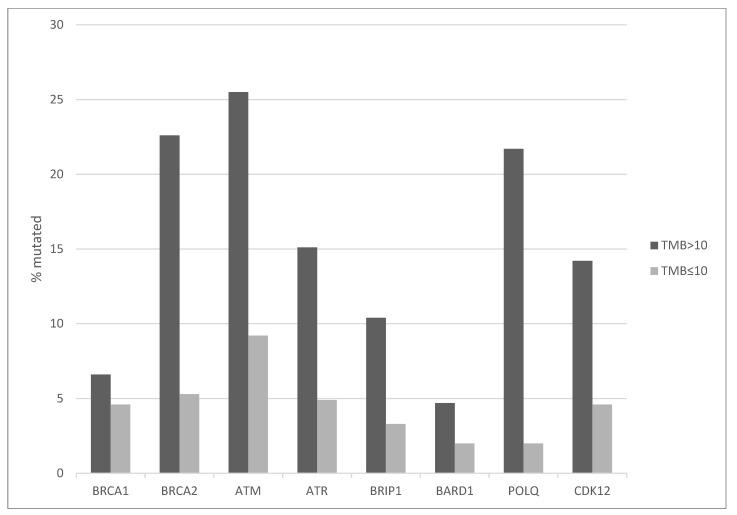
Prevalence of mutations in DNA Damage Response related genes in bladder carcinomas according to TMB groups. High TMB group: TMB > 10 mutations/MB, low TMB group: TMB ≤ 10 mutation/MB. TMB: Tumor Mutation Burden.

Mutations in DNA damage response genes are also observed more frequently in patients with high TMB, while they are rarer in the low TMB group (Figure 3). Mutations in *BRCA2*, *ATM* and *POLQ*, encoding for polymerase θ, are observed in more than 20% of cases each in the high TMB group and in 2% to 9.2% of cases in the low TMB group (Figure 3). Overall, 65.4% of cases in the high TMB group possess mutations in one or more of these DNA damage response genes while this percentage was 28.6% in the low TMB group (Fisher’s exact test *p* < 0.0001). A significant minority of these mutations, for example 18.9% for BRCA2 and 34.8% for ATM, are listed as likely oncogenic in the OncoKB knowledgebase. Table 3 shows the prevalence of likely oncogenic mutations in MMR and DNA damage response genes in the entire cohort and the two TMB groups.

Mutations in MMR associated genes, proofreading polymerases and DNA damage response genes occur independently from each other. Pairwise evaluation of comparison disclosed only four statistically significant co-occurrences, three of which involved POLQ mutations with MSH2, BRCA2 and CDK12 (Table 5).

**Figure 4:**
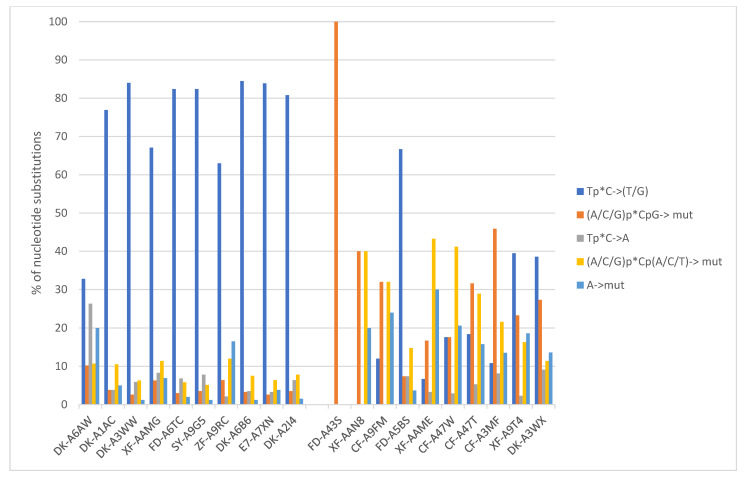
Base substitutions in representative samples with high TMB (left part of the figure) and samples with low TMB (right part of the figure). High TMB cases are dominated by signatures 2 and 13 (Tp*C > T/G) while low TMB cases are dominated by signature 5 (C > T/G/A). The identification code of evaluated cases is shown along the horizontal axis.

Mutation signatures that predominate in the group with high TMB include COSMIC SBS2 and SBS13 substituting a T or G respectively for a C at a position preceded by T (Figure 4). In contrast, cases in the low TMB group are dominated by signature SBS5, substituting C with any alternative base and associated with tobacco use.

**Figure 5:**
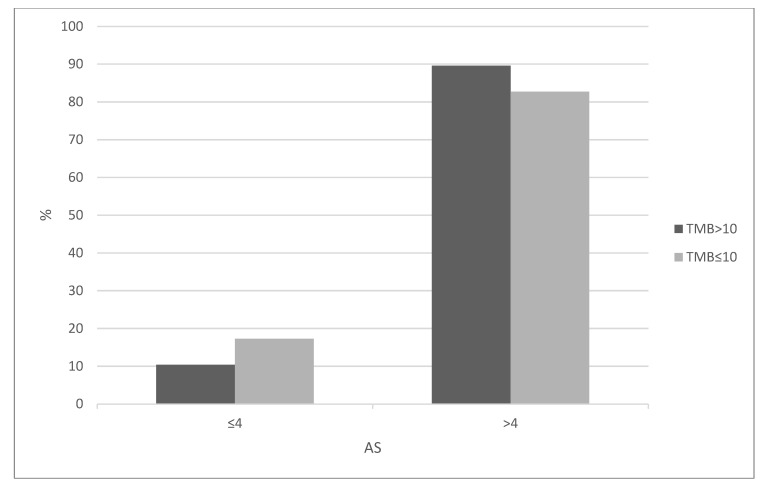
Aneuploidy Scores (AS) of above or below 4 in bladder carcinomas according to TMB. High TMB group: TMB > 10 mutations/MB, low TMB group: TMB ≤ 10 mutation/MB. Data are from TCGA. TMB: Tumor Mutation Burden.

**Figure 6:**
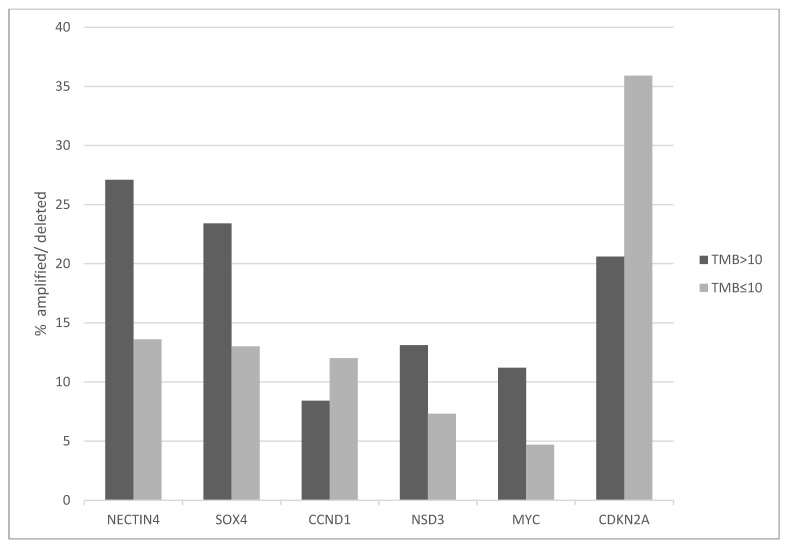
Common copy number alterations in bladder carcinomas according to TMB. High TMB group: TMB > 10 mutations/MB, low TMB group: TMB ≤ 10 mutation/MB. Data are from TCGA. TMB: Tumor Mutation Burden.

Chromosomal instability was high in bladder cancers with 84.5% of the cases in the cohort displaying an Aneuploidy Score (AS) above 4. The two groups did not differ on the number of tumors displaying chromosomal instability as measured by either an AS above 4 (Fisher’s exact test *p* = 0.11, Figure 5) or a similar metric, the Fraction of Genome Altered (Fisher’s exact test *p* = 0.11, not shown). However, there were differences between the frequency of individual copy number altered loci in the two groups. The most frequently amplified chromosomal loci in the whole bladder carcinoma cohort include locus 1q23.3, which encodes for *NECTIN4* and is amplified in 17.2% of cases, 6p22.3, which encodes for *SOX4* and *E2F3* and is amplified in 15.7% of cases, 11q13.3 which includes the locus of the gene encoding for cyclin D1, *CCND1* and is amplified in 11% of cases, and 8p11.23, which encodes for *NSD3* and *FGFR1* and is amplified in 8.8% of cases. The only common deletion concerns locus 9p21.3 which encompasses genes *CDKN2A* and *CDKN2B* encoding for tumor suppressors p14^ARF^ and p15^INK4b^ and occurs in 31.9% of bladder carcinomas. Deletions in 9p21.3 were more common in cancers with low TMB (Fisher’s exact test *p* = 0.003, Figure 6). Amplifications in the *NECTIN4* and the *SOX4* loci were more often observed in the high TMB group (Fisher’s exact test *p* = 0.002 and 0.01, respectively, Figure 6).

**Figure 7:**
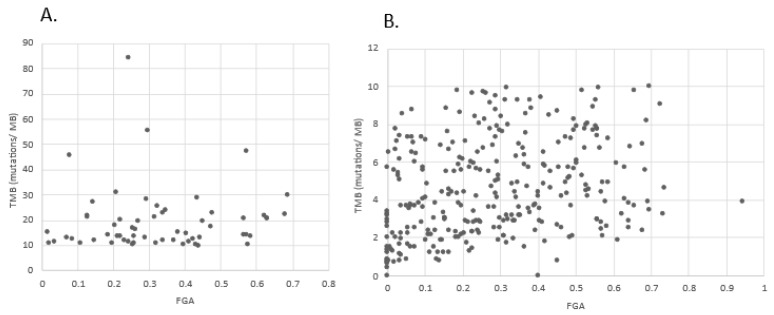
Correlation of TMB with chromosomal instability in: (**A**) The high TMB group (*n* = 107) and (**B**) The low TMB group (*n* = 298) of urothelial cancers in the TCGA cohort. FGA: Fraction Genome Altered.

There was no correlation of TMB with chromosomal instability as measured by the FGA in the high TMB group (Pearson’s R = 0.11, *p* = 0.24, Figure 7A). In contrast, a correlation of TMB with chromosomal instability was observed in the low TMB group (Pearson’s r = 0.29, *p* < 0.0001, Figure 7B). However, even in the low TMB group there is a broad level of chromosomal instability in all levels of TMB, as is evident from the figure.

**Figure 8:**
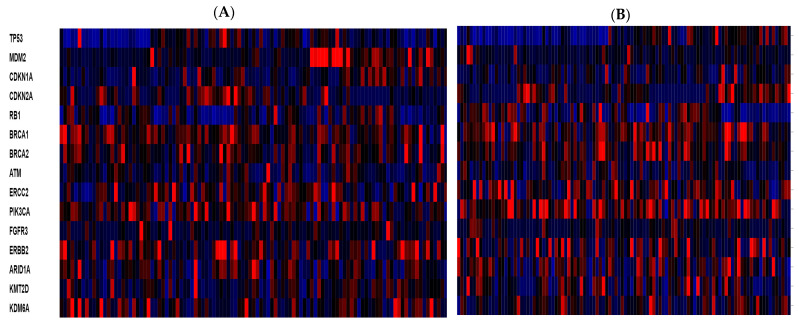
mRNA expression compared to diploid samples (RNA seq V2 RSEM) of key genes in bladder cancer pathogenesis in (**A**) cases with high TMB, and (**B**) representative cases with low TMB. Red denotes over-expression and blue under-expression.

At the transcriptomic level, genes with major roles in bladder carcinomas are expressed with high heterogeneity in the groups of cancers with high and low TMB. For example, p53 is down-regulated in a significant percentage of cases in both groups (Figure 8). An interesting observation pertains to tumor suppressor ubiquitin ligase MDM2 which is up-regulated in a sub-set of cases in the high TMB group but rarely in cases of the low TMB group.

**Figure 9:**
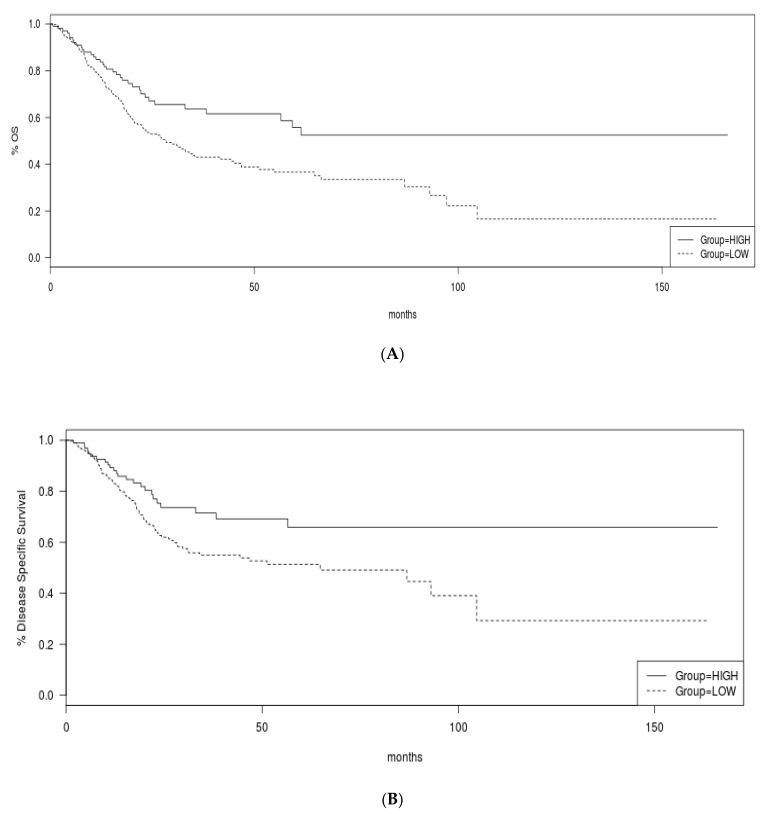
(**A**) Overall survival and (**B**) Disease Specific Survival of the high and low TMB groups.

**Figure 10:**
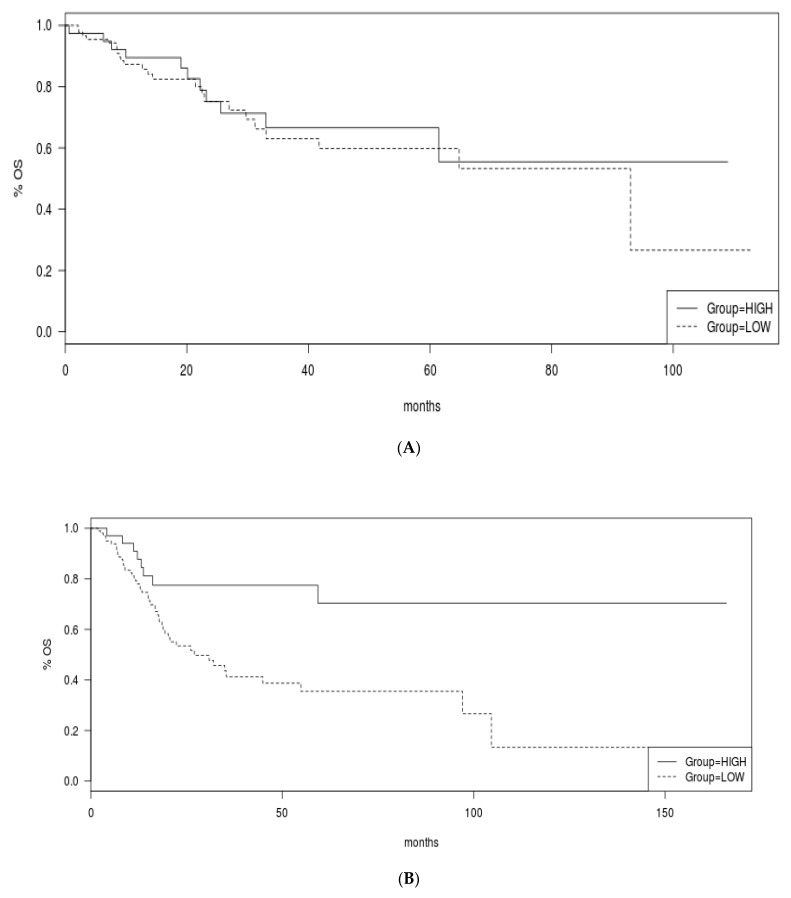

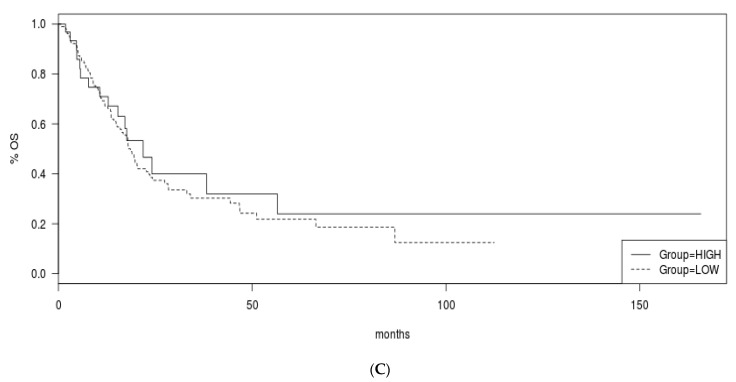
Overall Survival of the high and low TMB groups according to stage. (**A**) Stage II, (**B**) Stage III, (**C**) Stage IV.

Overall Survival (OS) was better in the group of urothelial bladder carcinomas with high TMB compared with the group with low TMB (Log Rank *p* = 0.002, Figure 9A). Similarly, the Disease Specific Survival was superior in the high TMB group (Log Rank *p* = 0.01, Figure 9B). Interestingly, the benefit of high TMB for OS was driven by the cohort with stage III disease while the two groups of patients with low and high TMB had similar OS when their cancers were of stage II or stage IV (Figure 10A–C).

## 4. Discussion

With the recent introduction of immunotherapy with checkpoint inhibitors in cancer therapeutics, a search for predictive biomarkers that can be used in the clinic to better determine responsive patients has followed. The total number of mutations that a cancer possesses, referred to as TMB, has been investigated along with expression of checkpoint molecule PD-L1 and the presence of Microsatellite Instability as predictive biomarkers [17]. The underlying premise for TMB is that increased mutations in a tumor lead to an abundance of neoantigen production for presentation to cytotoxic cells that are activated by exposure to immune checkpoint inhibitors [18]. In a phase 2 multicenter study of metastatic cancer patients with various cancers progressing on at least one line of chemotherapy, immunotherapy with the immune checkpoint inhibitor pembrolizumab resulted in objective responses (OR) in 29% of patients with high TMB as defined by at least 10 mutations per MB, compared with OR of 6% in patients with low TMB [19]. Given the association of high TMB with response to immunotherapy, the American FDA has granted approval of pembrolizumab for unresectable or metastatic tumors progressing to prior treatments with TMB ≥ 10 mutations/MB agnostic of primary site [10]. Increased TMB associated with increased immunogenicity is observed in tumor types that have been successfully treated with immune checkpoint inhibitors such as melanoma and non-small cell lung carcinomas [20,21]. Urothelial bladder carcinomas are also among the cancers that are successfully treated with immunotherapies [6,7]. Similar to other immunotherapy sensitive cancers, response is observed only in a minority of patients and biomarkers to determine patients destined to respond and to guide therapy are warranted [22]. The cut-off of 10 mutations/MB in the definition of immunotherapy sensitivity is artificial and the optimal cut-off may vary depending on the specific type of cancer [23]. A higher cut-off of 13 mutations/MB has been proposed as a better discriminator of immunotherapy response in tumors with overall high mutation burdens such as non-small lung cancer and melanoma [23]. TMB may in fact be predictive of immunotherapy response only in some types of cancers but not in others [24]. A study that analyzed neoantigen loads and CD8+ tumor infiltrating lymphocytes in various cancer types observed that there was a positive correlation only in some cancer types (so called type I: Lung adenocarcinoma, melanoma, bladder carcinomas, endometrial carcinomas) but no correlation in others (the so called type II cancers such as lung squamous carcinomas breast and prostate cancers). High TMB was predictive of response to immunotherapy only in type I cancers [24]. These data illustrate the fact that TMB is only one part of the interplay between cancer and the immune system, other parts played by antigen presentation, immune cell infiltration and immune cell activation. A study that examined a T cell inflamed Gene Expression Profile (GEP) in association with TMB across different datasets treated in pembrolizumab trials found that only a minority of bladder carcinomas with high TMB had a T cell inflamed GEP [25]. On the other hand, if other prerequisites are in place, some cancers with lower TMB may respond to immune checkpoint inhibitors, as observed in cases with low TMB but an inflamed GEP [25]. As a result, subsets of patients with various malignancies and low TMB do respond to immunotherapy [26].

In the current investigation genomic data from the TCGA bladder cancer cohort were interrogated aiming at defining the landscape of urothelial carcinomas with high TMB. In this cohort of 411 patients, 26% of the patients had a high TMB defined as more than 10 mutations per MB. Many but not all prevalent mutations in bladder carcinomas including *TP53, KMT2D*, *ARID1A*, *KMT2C*, *PIK3CA* and *EP300* are more commonly observed in the high TMB group compared with the low TMB group. Among less prevalent mutated genes with putative involvement in genomic instability, mutations in several MMR-related and DNA damage response genes are also more commonly observed in the high TMB group. In contrast, chromosomal instability is high in both groups, albeit with differences in individual altered loci. Bladder cancers with high TMB have a better prognosis than counterparts with low TMB. Interestingly, the improved prognosis seems to be driven exclusively by stage III cancers, while earlier stage II cancers and metastatic cancers have similar OS, independently of TMB.

These data do not necessarily support a direct causative role of mutations with increased prevalence, in cancers with high TMB, in the increase of mutation numbers, but suggest that increased TMB becomes better tolerated in their presence. Alternatively, some of these mutations may contribute to genetic instability. In this case, pathogenic mutations in driver genes are not direct triggers of the immune system as neoantigens but facilitate or contribute to the production of other mutations [27]. A combination of the two scenarios whence a driver mutation would increase genetic instability and in parallel would allow increased mutation tolerance through, for example, interference with antigen presentation, is also plausible and is supported by the fact that several prevalent molecular lesions are early events in bladder cancer pathogenesis [28,29]. Pathogenic mutations in the catalytic subunit alpha of kinase PI3K gene, *PIK3CA* is a case in point. Increased prevalence in high TMB has been described in other cancers [30,31]. An association of mutations in *PIK3CA* with mutations in MMR-related genes is also observed in these cancers. In addition, activating *PIK3CA* mutations or loss of its inhibitor phosphatase PTEN have been associated with decreased expression of Major Histocompatibility Cluster (MHC) I and II molecules leading to decreased antigen presentation [32].

In addition to the total number of new antigens depicted by the TMB, specific qualities and similarities of neoantigens produced in a cancer to antigens of pathogens are of importance for their ability to trigger an immune response [33]. Thus, the underlying mutation process is important to determine in tumors with a high TMB, and, indeed, even in tumors with lower TMB, to further inform the probability for immunotherapy responses [34]. Deaminases of the APOBEC family produce a specific mutation signature in cancers through deamination of cytosines [35]. Expression of two enzymes of the family APOBEC3A and APOBEC3B is associated with high TMB in bladder carcinomas [36]. Moreover, an APOBEC-high signature was associated with DNA damage response gene mutations and mutations in epigenetic regulators which, as shown in the current investigation, are also associated with high TMB [36]. Thus, molecular lesions underlying the APOBEC-high signature may be the culprit mutagenic process in some urothelial carcinomas with high TMB [37]. Urothelial carcinomas with the APOBEC-high signature harbor also pathogenic *PIK3CA* mutations at the hotspot helical domain amino-acid positions E542 and E545, which may underline the tolerance of these cancers to high TMB [36]. A relationship of the APOBEC mutation signature with *PIK3CA* E542K and E545K is also observed in HPV associated cervical and head and neck cancers [38]. Mutations in DNA damage response genes, such as *BRCA1* and *BRCA2*, observed in breast cancer are also associated with production of elevated mutation numbers, particularly of strong binder peptides to MHC molecules [39]. Despite this, absence rather than presence of such mutations segregated with tumor immunogenicity, suggesting that the increased TMB leads to pressure for selection of cells with defective antigen presentation or up-regulation of immune checkpoints. Antigen presentation defects in MMR-related or proofreading polymerase mutated cancers may underpin resistance to checkpoint inhibitors and be the cause of lack of response in a significant number of MMR deficient cancers in the clinic [40,41].

ERCC2 is a protein participating in nucleotide excision repair (NER) and is mutated in a subset of hypermutated bladder carcinomas. Genetic NER defects lead to the xeroderma pigmentosum disease spectrum with skin cancer predisposition. ERCC2 mutations in bladder cancer are linked with current smoking status [42]. In addition, xeroderma pigmentosum-associated tumors present with increased tumor burden and may be sensitive to checkpoint inhibitors [43].

High frequency of mutations in epigenetic enzymes has been previously described in urothelial carcinomas [44,45]. The higher prevalence of epigenetic modifier mutations in bladder carcinomas with high TMB is interesting from a therapeutic point of view. Mutations of histone acetyltransferase EP300 are linked to a hyperacetylated state and tumor sensitivity to radiation therapy, through acetylation of BRCA1 promoter-associated histone 3 at lysine 27 (H3K27) and repression of homologous recombination repair [46]. In bladder carcinoma therapy, radiation is commonly used in conjunction with cisplatin, a DNA damaging agent which may further potentiate creation of double strand lesions that would be accumulating in cancers with epigenetic BRCA1 neutralization. In TCGA bladder cohort, EP300 and BRCA1 mutations are present in 15.4% and 5.1% of cases, respectively, but only three cases have concomitant mutations, suggesting that the two genes may be part of the same pathway. Another study has suggested that some of the mutations of the histone acetyltransferase domain in EP300 and in the homologous acetyltransferase CREBBP encountered in bladder carcinoma patients would to reduction of their function and could be counteracted by treatment with histone de-acetylase inhibitor mocetinostat [44]. An in vitro investigation used the epigenetic modifier PLX51107, a BET (Bromodomain and Extra-Terminal motif) inhibitor, to successfully sensitize bladder carcinoma cells to platinum and PARP inhibitor talazoparib [47]. BET inhibitors interfere with the function of proteins that read the histone acetylation signal and are downstream of abnormal acetylation. BET inhibitors have had limited success as monotherapies in cancer treatment but development in combination with other targeted agents or immunotherapy in carefully selected patients with the appropriate genetic lesions may rekindle interest in the field [48,49]. For example, one could envision a trial of a combination of a checkpoint inhibitor with a BET inhibitor in bladder cancers with high TMB and EP300 mutations, while patients with high TMB and DNA damage response defects could be assigned to a checkpoint inhibitor with PARP inhibitor arm.

The current study is based on an extensive cohort of bladder carcinoma patients investigated by whole exome sequencing in a multi-institutional effort under the umbrella of TCGA. The analysis of TMB reflects the platform used in the original data and may not be directly transferable to TMB calculated by other platforms. Moreover, as mentioned above, the cut-off of 10 mutations/MB may not be the clinically optimal cut-off, although it is currently that used in the clinic. There are additional nuances associated with the specific type of mutations that constitute the total burden, which lead to differential antigen presentation and are not captured by the row TMB as a biomarker for immunotherapy response. A next generation of computational efforts based on mutational signatures capturing nucleotide substitution heterogeneity, as well as structural prediction of strong antigen presentation depending on the specific patient HLA phenotype, are going to revolutionize the field and may lead to further promotion of a personalized approach [50,51].

In conclusion, therapeutic opportunities arise from the delineation of the molecular landscape of bladder carcinomas with high TMB that deserve further exploration, possibly in combination with chemotherapy or immunotherapies. These could include combinations with PI3K pathway inhibitors, PARP inhibitors and histone de-acetylase as well as other epigenetic inhibitors in trials of genomically selected sub-sets of patients. Some of these studies are planned or in early development [52,53].

## Figures and Tables

**Table 1:** Characteristics of patients in the whole TCGA cohort of urothelial bladder carcinoma and according to the TMB groups. NA: Not available.

Characteristic	Entire Group (*n* = 411) (%)	TMB >10 (*n* = 107)	TMB ≤ 10 (*n* = 304)	*p*
Age (mean ± SD)	68.1 ± 10.6	67.7 ± 9.6	68.3 ± 10.9	0.6
Age				
≤65 years-old	161 (39.2)	42 (39.3)	119 (39.1)	1.0
>65 years-old	250 (60.8)	65 (60.7)	185 (60.9)	
Sex				
Male	303 (73.7)	85 (79.4)	218 (71.7)	0.12
Female	108 (26.3)	22 (20.6)	86 (28.3)	
stage				
I	2 (0.5)	0	2 (0.7)	0.5
II	130 (31.6)	38 (35.5)	92 (30.3)	
III	141 (34.3)	37 (34.6)	104 (34.2)	
IV	136 (33.1)	32 (29.9)	104 (34.2)	
NA	2 (0.5)		2 (0.7)	

**Table 2:** Functional significance of commonly mutated genes in urothelial bladder carcinoma in the whole cohort and the groups with high and low TMB. The table shows the number and percentage (in parentheses) of mutations evaluated as likely oncogenic in the OncoKB knowledgebase.

Gene	Entire Group (%)	TMB > 10 (%)	TMB ≤ 10 (%)	*p*
TP53	235/237 (99.2%)	77/79 (97.5%)	158/158 (100%)	-
KMT2D	106/163 (65%)	39/69 (56.5%)	67/94 (71.3%)	0.07
ARID1A	92/134 (68.7%)	33/49 (67.3%)	59/85 (69.4%)	0.84
PIK3CA	77/99 (77.8%)	26/35 (74.3%)	51/64 (79.7%)	0.61
KMT2C	51/110 (48.2%)	25/64 (39.1%)	26/46 (56.5%)	0.08
EP300	36/81 (44.4%)	14/41 (34.1%)	22/40 (55%)	0.08

**Table 3:** Functional significance of genes involved in MMR and DNA damage response in urothelial bladder carcinoma in the whole cohort and the groups with high and low TMB. The table shows the number and percentage (in parentheses) of mutations evaluated as likely oncogenic in the OncoKB knowledgebase. The remaining mutations, except for one PMS2 mutation predicted to be neutral, are of unknown significance.

Gene	Entire Group (%)	TMB > 10 (%)	TMB ≤ 10 (%)	*p*
MSH2	4/13 (30.8%)	4/10 (40%)	0/3	-
MSH6	2/10 (20%)	2/8 (25%)	0/2	-
PMS2	1/10 (10%)	0/4	1/6 (16.7%)	-
MLH1	3/9 (33.3%)	1/5 (20%)	2/4 (50%)	-
POLE	1/26 (3.8%)	1/10 (10%)	0/16	-
POLD1	0/10	0/4	0/6	-
BRCA1	7/24 (29.2%)	2/7 (28.6%)	5/17 (29.4%)	1
BRCA2	10/53 (18.9%)	5/37 (13.5%)	5/16 (31.2%)	0.14
ATM	24/69 (34.8%)	11/39 (28.2%)	13/30 (43.3%)	0.21
ATR	3/36 (8.3%)	2/19 (10.5%)	1/17 (5.9%)	1
BRIP1	6/23 (26.1%)	1/11 (9.1%)	5/12 (41.7%)	0.15
BARD1	1/11 (9.1%)	0/5	1/6 (16.7%)	-
POLQ	0/31	0/25	0/6	-
CDK12	6/32 (18.7%)	3/15 (20%)	3/17 (17.6%)	1

**Table 4:** Hypermutated cases with TMB ≥ 30 mutations/MB. The third column presents the total number of mutations in each case estimated as likely oncogenic in the OncoKB knowledgebase and the fourth column presents examples of genes with likely oncogenic mutations.

Patient ID	TMB (Mutations/MB)	Total Likely Oncogenic in OncoKB	Mutations
TCGA-DK-A6AW	118.6	29	POLE, MSH2, CTCF, ARID1A, KMT2C, KMT2D, STAG2, ATM
TCGA-KQ-A41N	84.6	33	ATM, ATR, CTCF, ERCC2, FAT1, ARID1A
TCGA-K4-A54R	55.8	12	PIK3CA, FAT1, ARID1A
TCGA-MV-A51V	47.6	6	ARID1A, KMT2C, STAG2
TCGA-YC-A89H	45.7	18	ERCC2, ARID1A, PIK3CA, KMT2C, KMT2D, APC
TCGA-DK-A1AC	43.2	13	ERCC2, PIK3CA, FANCD2
TCGA-DK-A3WW	36.1	16	PIK3CA, ATM, KMT2C, KMT2D
TCGA-BT-A2LB	34.4	9	APC, KMT2A, ARID2
TCGA-SY-A9G5	31	12	PIK3CA, ARID1A, KMT2A, ARID2
TCGA-XF-AAMG	30	11	PIK3CA, ATM, KDM6A, KMT2D

**Table 5:** Statistically significant co-occurrences between mutations in MMR associated genes, proofreading polymerases and DNA damage response genes in the whole TCGA bladder carcinoma cohort. Correction for multiple comparisons was performed with the Benjamini-Hochberg false discovery rate procedure.

Co-Occurring Genes	Number of Cases with Co-Occurrence (%)	q
BRCA2 ATM	14 (3.4%)	0.02
POLQ MSH2	5 (1.2%)	0.03
POLQ BRCA2	9 (2.2%)	0.03
POLQ CDK12	7 (1.7%)	0.03

## Data Availability

Not applicable.
